# Distinct thermodynamic strategies in cello-oligosaccharide binding by *Anaerocellum bescii* substrate-binding proteins

**DOI:** 10.1128/aem.00511-26

**Published:** 2026-06-17

**Authors:** Jonghoon Kang

**Affiliations:** 1 Department of Biology, Valdosta State University 15614, Valdosta, Georgia, USA; University of Nebraska-Lincoln, Lincoln, Nebraska, USA

**Keywords:** cellulose, cello-oligosaccharide, ABC sugar transporter, substrate-binding protein, enthalpy, entropy, thermodynamics

## LETTER

I read with great interest the recent article by Tjo et al. describing the biophysical and genetic characterization of a conserved ATP-binding cassette transporter mediating cello-oligosaccharide uptake in *Anaerocellum bescii* (1). The integration of isothermal titration calorimetry, structural modeling, and targeted gene deletion provides important mechanistic insight into carbohydrate uptake in this extremely thermophilic, lignocellulolytic bacterium.

The authors of the original article (1) report complete thermodynamic parameters (Δ*H*, Δ*S*, and Δ*G*) for the binding of cello-oligosaccharides to the substrate-binding proteins (SBPs) Athe_0597 and Athe_0598. These data permit an additional thermodynamic analysis not explicitly discussed in the article—namely, enthalpy-entropy compensation (EEC), an extra-thermodynamic phenomenon in which coordinated changes in Δ*H* and Δ*S* limit variation in Δ*G* across related reactions (2, 3). Although EEC is widely observed in protein-ligand systems, examining such relationships within a defined ligand series can provide mechanistic insight into how substrate-binding proteins maintain recognition across chemically related substrates (4–7) and therefore offers an additional interpretive perspective on the thermodynamic data set reported by Tjo et al. Here, I reanalyze the thermodynamic data set reported by Tjo et al. to determine whether compensation behavior is present in the binding interactions of the substrate-binding proteins Athe_0597 and Athe_0598.

**Fig 1:**
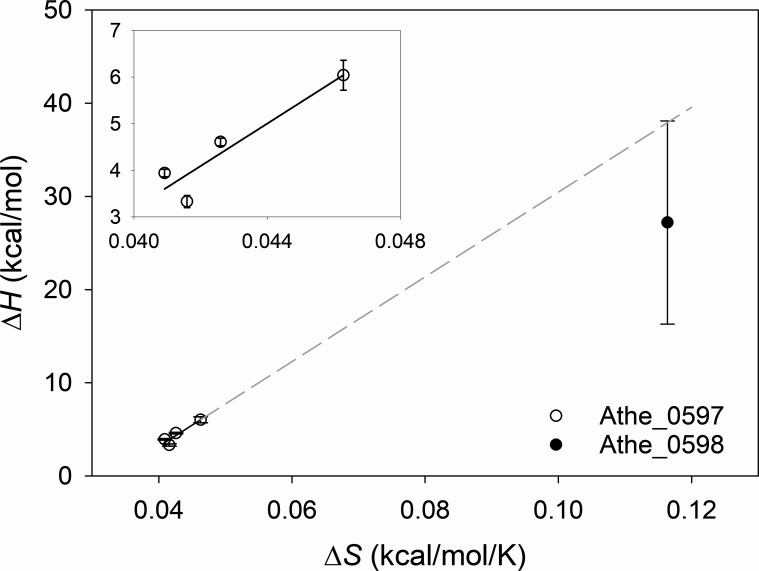
Linear relationship between binding enthalpy (Δ*H*) and entropy change (Δ*S*) demonstrating enthalpy-entropy compensation in the Athe_0597 ligand series (open circles; cellobiose-cellopentaose). The filled circle represents the Athe_0598–cellobiose interaction. The solid line represents the linear regression for the Athe_0597 data set according to Δ*H* = *T_c_*Δ*S* + β, yielding *T_C_* = 455 ± 122 K and β = –15 ± 5 kcal/mol (*R*^2^ = 0.875). The dashed line shows extrapolation of this fit for comparison. Error bars indicate reported uncertainties in Δ*H*. The inset shows the Athe_0597 data on an expanded scale.

Figure 1 shows the relationship between Δ*H* and Δ*S* for the reported binding interactions. When the Athe_0597 ligand series (cellobiose-cellopentaose) is considered independently, Δ*H* and Δ*S* display a linear relationship described by Δ*H* = *T_c_*Δ*S* + β, where *T_c_* is the compensation temperature (slope) and β is the intercept (8, 9). This pattern is consistent with classical EEC, in which enthalpic penalties are offset by favorable entropic contributions. Such behavior is widely observed in protein-ligand systems and helps explain robust ligand recognition within a conserved binding scaffold (4, 5).

To quantify the relative variability of the thermodynamic parameters, the coefficients of variation (CV) were calculated (10). Within the Athe_0597 ligand series, Δ*G* varies substantially less than Δ*H*, and the CV for *T*Δ*S* is similarly low. *T*Δ*S* and Δ*G* were calculated at 78°C, corresponding to the optimal growth temperature of *A. bescii* (11). The CV values for Δ*G*, Δ*H*, and *T*Δ*S* are 0.045, 0.26, and 0.056, respectively. This limited variation in Δ*G* relative to Δ*H* is consistent with EEC.

By comparison, the Athe_0598-cellobiose interaction occupies a different energetic regime, exhibiting substantially larger magnitudes of Δ*H* and Δ*S* than those observed for the Athe_0597 ligand series (Fig. 1). Because thermodynamic measurements for Athe_0598 are currently available for only a single ligand (1), it is not possible to determine whether a comparable compensation relationship exists for this SBP. Accordingly, the available data do not permit a statistical assessment of EEC for Athe_0598. When the Athe_0598–cellobiose interaction is included in a combined regression with the Athe_0597 data set, regression diagnostics indicate that this point is highly influential (Cook’s distance far exceeding the conventional threshold of 4/*n*, where *n* is the number of observations), reflecting its extreme leverage relative to the Δ*S* range defined by the Athe_0597 ligand series (12). For this reason, the compensation analysis was restricted to the Athe_0597 ligand series (Fig. 1). Nevertheless, the large energetic magnitudes observed for the Athe_0598-cellobiose interaction suggest that ligand recognition in this SBP may involve a different thermodynamic partitioning compared with Athe_0597. Additional ligand-binding measurements would be required to determine whether this difference reflects a systematic energetic strategy or simply ligand-specific effects.

Together, the data support two complementary conclusions. First, the Athe_0597 ligand series displays ligand length-dependent EEC consistent with a compensatory binding strategy. Second, the thermodynamic profile of the Athe_0598-cellobiose interaction differs markedly from the Athe_0597 trend, suggesting that ligand recognition in this SBP may involve a different energetic partitioning. This interpretation is consistent with structural differences reported for the two SBPs, although additional binding measurements will be required to determine whether this difference represents a systematic energetic strategy. Recognition of this compensation behavior provides a thermodynamic framework for understanding how *A. bescii* maintains efficient uptake across chemically related substrates and illustrates how related SBPs within a single transporter system may employ different energetic strategies for carbohydrate recognition.
